# Two *Leucobacter* Strains Exert Complementary Virulence on *Caenorhabditis* Including Death by Worm-Star Formation

**DOI:** 10.1016/j.cub.2013.08.060

**Published:** 2013-11-04

**Authors:** Jonathan Hodgkin, Marie-Anne Félix, Laura C. Clark, Dave Stroud, Maria J. Gravato-Nobre

**Affiliations:** 1 Department of Biochemistry, University of Oxford, Oxford OX1 3QU, UK; 2 Institute of Biology, Ecole Normale Supérieure, CNRS-Inserm, 75230 Paris Cedex 05, France

## Abstract

The nematode *Caenorhabditis elegans* has been much studied as a host for microbial infection. Some pathogens can infect its intestine [1, 2], while others attack via its external surface [1, 3–6]. Cultures of *Caenorhabditis* isolated from natural environments have yielded new nematode pathogens, such as microsporidia and viruses [7, 8]. We report here a novel mechanism for bacterial attack on worms, discovered during investigation of a diseased and coinfected natural isolate of *Caenorhabditis* from Cape Verde. Two related coryneform pathogens (genus *Leucobacter*) were obtained from this isolate, which had complementary effects on *C. elegans* and related nematodes. One pathogen, Verde1, was able to cause swimming worms to stick together irreversibly by their tails, leading to the rapid formation of aggregated “worm-stars.” Adult worms trapped in these aggregates were immobilized and subsequently died, with concomitant growth of bacteria. Trapped larval worms were sometimes able to escape from worm-stars by undergoing autotomy, separating their bodies into two parts. The other pathogen, Verde2, killed worms after rectal invasion, in a more virulent version of a previously studied infection [6]. Resistance to killing by Verde2, by means of alterations in host surface glycosylation, resulted in hypersensitivity to Verde1, revealing a trade-off in bacterial susceptibility. Conversely, a sublethal surface infection of worms with Verde1 conferred partial protection against Verde2. The formation of worm-stars by Verde1 occurred only when worms were swimming in liquid but provides a striking example of asymmetric warfare as well as a bacterial equivalent to the trapping strategies used by nematophagous fungi [4].

## Highlights

• Verde1 bacteria can trap swimming worms by tail adhesion and worm-star formation
• Nematodes trapped in worm-stars are immobilized, killed, and used for nutrition
• Trapped larval worms can escape from worm-stars by whole-body autotomy
• Resistance to virulent Verde2 bacteria results in lethal hypersensitivity to Verde1

## Results and Discussion

Cultures of *Caenorhabditis* collected from many locations have been examined for the presence of a swollen tail or “Dar” (*d*eformed *a*nal *r*egion) phenotype, which is the conspicuous morphological response of some rhabditid nematodes to rectal infection [6, 9]. This response was discovered in laboratory stocks fortuitously infected by a coryneform bacterium, *Microbacterium nematophilum*, which has been repeatedly isolated in nematode laboratories [6, 10], but never from nature. Two recent *Caenorhabditis* isolates were found to exhibit a Dar phenotype, which proved to carry *Leucobacter* infections with distinctive pathogenic effects.

**Figure 1:**
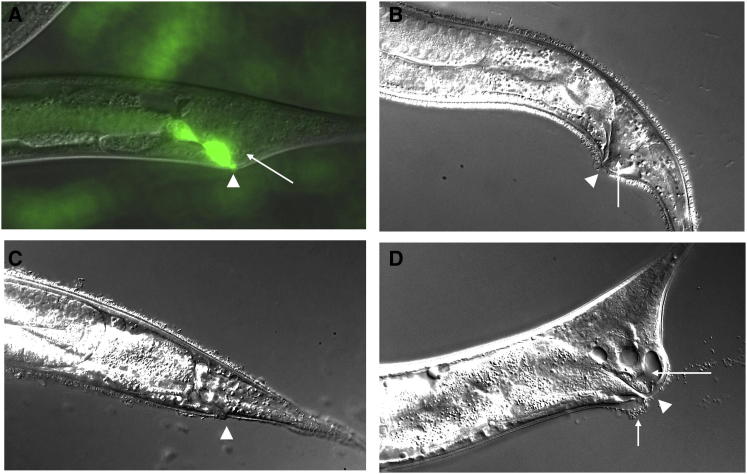
*Leucobacter* Cells Adhere to *Caenorhabditis* (A) Adult *C. elegans* hermaphrodite, infected with *Leucobacter* CBX130 and vitally stained with SYTO13 (green fluorescence). Rectal infection and tail swelling (arrow) are apparent. (B) Tail region of strain JU1635. Note tail swelling (arrow) and extensive bacterial coating. (C) Tail region of *C. elegans* infected with Verde1. Note extensive bacterial coating and absence of tail swelling. (D) Tail region of *C. elegans* infected with Verde2. Note severe tail swelling (arrow) and bacteria attached only around anus (short arrow). Arrowhead marks anal opening in (A)–(D).

One *C. elegans* isolate (JU1088), collected in Kakegawa, Japan, exhibited poor growth, swollen tails, and visibly adherent bacteria. A bacterial strain, CBX130, was obtained from these infected worms and was found to cause pathogenic effects similar to *M. nematophilum* when added to cultures of the *C. elegans* laboratory strain N2 (Figure 1A). 16S rRNA gene sequencing indicated that CBX130 belonged to the coryneform genus *Leucobacter*.

A second Dar isolate (JU1635), obtained from rotting banana trunks in Cape Verde, was found to be a hermaphrodite species of *Caenorhabditis* (n. sp. 11) [11]. Worms in this population had swollen tails as well as a dense covering of adherent bacteria over the surface of the body (Figure 1B). A similar disease state was established in *C. elegans* (N2) by growing worms on bacterial lawns that had been exposed to JU1635. Infected *C. elegans* worms had the dense bacterial coating and swollen tails seen in the original JU1635 population.

Isolation of bacteria from the diseased *C. elegans* population revealed that worms carry a double infection of two distinct bacteria with contrasting pathogenic effects (Figures 1C and 1D). Two pathogenic strains were established, both typed as *Leucobacter* species. We refer to them here as Verde1 and Verde2; detailed phenotypic and taxonomic characterization will be presented elsewhere. Verde1 is identical in 16S RNA sequence to *Leucobacter celer* NAL101 [12], though distinguishable from it (see Table S1 available online), whereas Verde2 is identical in 16S RNA sequence to the Kakegawa isolate CBX130. These two bacteria had remarkably different effects on *C. elegans*. Both Verde1 and Verde2 were virulent pathogens, but they exerted their lethality in distinct ways.

Under standard culture conditions, Verde1 was inhibitory but not lethal to the propagation of wild-type *C. elegans*. Worms grown on mixed lawns of *E. coli* and Verde1 (∼10:1) became impaired in movement and grew more slowly (generation time increased by 27%; Table S2), but they were able to grow and reproduce. When examined microscopically, infected worms were seen to have a dense covering of tightly adherent bacteria, but no Dar phenotype (Figure 1C; Figure S1).

**Figure 2:**
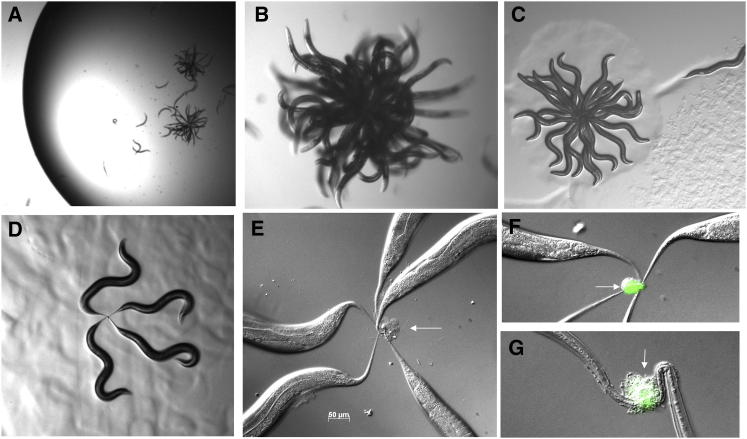
Verde1 Bacteria Induce Formation of Worm-Stars (A) Two worm-stars forming in 0.2 ml drop. Most of the worms in the drop have been captured by the stars. (B) Close-up of a worm-star in liquid. (C) A worm-star after transfer to a solid surface; one worm can be seen escaping. (D) Snapshot of a quartet of worms held together by their tails. (E) Five worms held together by a tail knot (arrow). (F) A four-worm tail knot induced by Verde1 bacteria that had been prelabeled with SYTO13; green fluorescence (arrow) is confined to the tail-spike region. (G) Detail of tail adhesion between two worms; fluorescent adherent bacteria can be seen (arrow).

**Figure 3:**
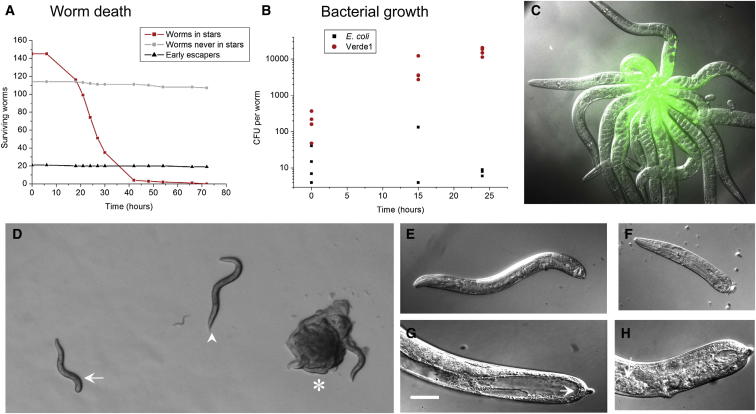
Worm Killing and Escape by Autotomy (A) Survival of worms in stars. Ten small stars were allowed to form in liquid from about 300 young adult worms and then picked to agar plates. Worms that avoided stars or that escaped in the first hour after picking were collected separately and transferred to agar plates. Plates were incubated at 25°C, and worm viability was scored over time. (B) Bacterial counts in small worm-stars over time, on nutrient-free agarose. (C) A 15-worm-star after 8 hr of incubation at 25°C, stained briefly with fluorescein diacetate; epifluorescence/differential interference contrast optics. (D) Remains of an L4 worm-star (asterisk) 20 hr after formation, showing one escaper with a truncated tail spike (arrowhead) and one half-worm escaper (arrow). (E) A 60% half-worm. (F) A 40% half-worm. (G) Half-worm posterior, with blind gut ending (arrow). Scale bar represents 20 μm. (H) Half-worm posterior, with developing egg (arrow) anterior to vulva.

Exposure of worms to Verde1 bacteria in liquid had different consequences. Within 2 min after addition of a Verde1 bacterial suspension (∼10^8^ colony-forming units [cfu]/ml) to a population of worms swimming in liquid, they began to stick to each other in their tail regions and formed star-like aggregates, which we term “worm-stars,” with dozens of worms radiating outward from a posterior zone of adhesion (Figures 2A and 2B). When the aggregates were picked out of liquid onto an agar plate, the trapped worms tried to escape from the stars, but most (>80%) of them failed (Figure 2C). Trapping had lethal consequences: after 24–48 hr incubation at 25°C, all the worms in a star were dead (Figure 3A). The worms that escaped from a star within the first hour survived for much longer, but worms that broke free at later times moved only a few body lengths from the star before dying. Worms that avoided trapping into stars could be similarly transferred from liquid onto solid media, and these showed no major ill effects and survived for many days (Figure 3A).

Death of worms in stars appeared to result primarily from destruction of permeability barriers in the tail of the worms. Loss of integrity in the posterior of worms was demonstrated by staining worm-stars with fluorescein diacetate, which could be seen diffusing into body cavities of the trapped worms from the tail forward (Figure 3C).

Stars containing few worms formed if numbers were kept low, and these were picked out and examined. Individuals were seen to be tied together by a knot formed by their interwoven tail spikes (Figure 2D–2G). Small stars sometimes disassembled, by unweaving or breakage of tail spikes, but the larger the aggregate, the more irreversible the knot. Star formation began within 2 min at high bacterial concentrations (>10^8^ cfu/ml) but still occurred down to 10^5^ cfu/ml, albeit more slowly. At these low concentrations, only a few thousand bacterial cells sufficed to stick worms together. Inducing the formation of worm-stars with prelabeled fluorescent Verde1 bacteria showed that the bacteria were initially preferentially attaching to worm tails (Figures 2F and 2G).

Under these liquid conditions, the bacteria were therefore waging an ingenious form of asymmetric warfare. Despite being vastly smaller than their hosts, they could immobilize substantial groups of worms, which could then presumably be degraded and used for bacterial nutrition. Cuticular damage and bacterial invasion were probably exacerbated by the worms’ continued writhing during efforts to escape, as well as tail breakage in worms that succeeded in breaking free.

Pathogenic proliferation was demonstrated by measuring the number of bacteria in worm-stars (Figure 3B). Small stars were allowed to form, washed, and sampled for total bacterial content shortly after formation or after 15 or 24 hr of incubation. Initial counts averaged ∼200 Verde1 bacteria per worm, whereas 24 hr counts averaged ∼16,000, revealing an 80-fold increase. Residual *E. coli* counts dropped from an average of 17 to 6 per worm over the same time, demonstrating that Verde1 dominated bacterial growth under these conditions.

Adult individuals that escaped from worm-stars often displayed broken tail spikes that had healed, consistent with wound repair [3]. Another defensive response was observed when worm-stars formed from populations of late larval (L4) worms. 24 hr after star formation, about 5% of the worms in such stars (7 of 82 worms in six stars) had escaped by undergoing a form of autotomy, whereby the worm became separated into two parts (Figures 3D–3F). The resulting anterior half-worms were able to crawl away from the degrading worm-stars. Gonads in half-worms had often undergone sufficient maturation to allow self-fertilization and progeny production (Figure 3F). Worms that had separated anterior to the vulva rarely produced progeny (1 of 11 individuals examined), but those with more posterior autotomy were usually fertile (42 of 53 individuals examined, each producing one to five progeny). Consequently, autotomy represents a viable escape strategy in terms of reproductive survival, though it was not seen in adult worms. Autotomy has not been reported previously in nematodes [13].

Verde1 was observed to form worm-stars in the original *Caenorhabditis* strain JU1635 and in other tested species of *Caenorhabditis* (Figure S2). Verde1 also induced stable star formation in nematodes from some related nematode genera, such as *Oscheius tipulae* and *Rhabditis brassicae*, but not in *Pristionchus pacificus*, for which only weakly bound stars and loose tangles formed. When populations of *P. pacificus* were mixed with *C. elegans* and exposed to Verde1 in liquid, stable stars containing both species were formed (Figure S2), with subsequent death of both constituents. The ability to trap and kill less susceptible species such as *P. pacificus* by means of mixed-species stars enlarges the potential host range of Verde1. Worm-stars were also observed to form under simulations of natural conditions, such as when rain falls on rotting fruit, a preferred habitat for *C. elegans* [7, 11, 14] (Figure S2).

**Figure 4:**
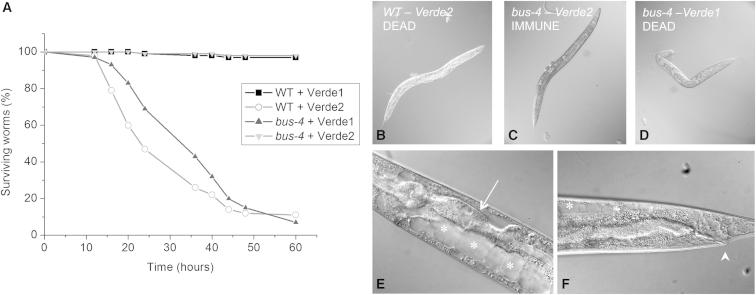
Verde1 and Verde2 Exert Complementary Lethal Effects (A) Survival of wild-type or *bus-4(e2693)* mutant worms on *E. coli*/Verde1 or *E. coli*/Verde2 lawns at 25°C. For each condition, 100 L4 worms were picked and examined for viability over time. (B) Wild-type worm and (C) immune *bus-4* worm after 24 hr incubation on *E. coli*/Verde2. (D) *bus-4* worm after 24 hr incubation on *E. coli*/Verde1. Note transparency, shrinkage, and collapse relative to (C). (E and F) Higher-magnification details of wild-type worms dying after 20 hr exposure to Verde2. In (E), note the anterior gut (arrow) compressed by swollen vacuoles (asterisks) in the body cavity. Vacuoles are also visible in the tail region (F), as well as bacteria adhering to anal cuticle (arrowhead).

The other Cape Verde *Leucobacter*, Verde2, had different effects on *C. elegans*. Worms grown at 20°C or below in the presence of Verde2 developed a Dar phenotype, similar to that caused by *M. nematophilum* and CBX130 (Figure 1D). At higher temperatures, Verde2 was more virulent. Young adult worms exposed to mixed lawns of *E. coli*/Verde2 at 25°C mostly died within 36 hr (Figure 4A), and larvae were almost wholly unable to survive. Rare escapers had extremely swollen tails and usually failed to reach adulthood. Verde2 is therefore a lethal pathogen for *C. elegans*, with effects similar to but more virulent than those of *M. nematophilum*.

Adult worms killed by Verde2 exhibited an unusual rigid and swollen appearance (Figure 4B). Microscopic examination revealed that the body cavity of dying worms became filled with large vacuoles, which distorted internal organs and distended the whole body (Figures 4E and 4F). General internal lysis ensued. How the Verde2 pathogen is able to exert this effect is mysterious.

Similarity between Verde2 infection and *M. nematophilum* infection was seen in the response of numerous *C. elegans* mutants selected for altered response to *M. nematophilum* in previous work [15, 16]. Most of the mutants resistant to infection by *M. nematophilum* were also fully resistant to Verde2, and consequently they were able to grow well on *E. coli*/Verde2 lawns (Figure 4; Figure S3; Table S3). Among these were many mutants with altered surface glycosylation, which have been extensively described (*bus* and *srf* mutants [16–20]).

When these Verde2-resistant glycosylation mutants were exposed to Verde1, it was found that the Verde1 pathogen efficiently killed them, so populations of Bus or Srf worms were completely unable to survive on mixed *E. coli*/Verde1 lawns (Figure 4; Figure S3; Table S3). Death appeared to result from general cuticular destruction and loss of surface integrity, because Bus adults killed by Verde1 had a collapsed translucent appearance (Figure 4D), in contrast to the straight engorged corpses resulting from Verde2 infection of wild-type adults (Figures 4B, 4E, and 4F).

Verde1 therefore has latent potential as a virulent pathogen for *C. elegans*. The response of wild-type *C. elegans* to Verde1 infection was examined using the induction of the defensive antimicrobial peptide NLP-29 [3]. After 8 hr of exposure to Verde1, quantitative RT-PCR measurement showed that expression of *nlp-29* in wild-type worms was increased by a factor of 6.8 ± 2.9. Similarly, the reporter strain IG274, which expresses GFP driven by the *nlp-29* promoter [3], revealed pathogenic induction of epidermal *nlp-29p::gfp* expression after exposure to Verde1 (Figure S4).

The reciprocal relationship between Verde1 and Verde2 pathogenicity reveals a trade-off in susceptibility: worms cannot become resistant to Verde2 or related pathogens without becoming lethally hypersensitive to Verde1. The presence of both bacteria on the original sample of naturally infected worms, strain JU1635, raised the possibility that double infection might be better tolerated than single infections. Under some circumstances, preinfection by Verde1 was found to be protective against subsequent challenge by Verde2. This was demonstrated by briefly exposing young adult wild-type *C. elegans* to Verde1 in liquid conditions (for one minute, too short a time for the formation of worm-stars), followed by plating on *E. coli*/Verde2 lawns. After 24 hr incubation at 25°C, 73% of control worms (147 of 200) were dead, while only 43% of Verde1-treated worms (86 of 200) were dead. The tolerance of Verde2 infection in the original JU1635 sample was probably further increased by the presence of additional bacterial strains in this culture (Table S2). Verde2 did not interfere with star formation by Verde1 but rather even promoted it (Table S4).

These two pathogens, obtained from the same wild isolate of *Caenorhabditis*, therefore exhibited diverse effects on *C. elegans*. Verde1 was capable of killing worms by means of star formation under liquid conditions but was tolerated and mildly protective against Verde2 when worms were growing on solid substrates. In contrast, Verde2 caused severe rectal disease and lethality to wild-type worms, while mutant worms resistant to Verde2 by virtue of surface alteration became lethally hypersensitive to Verde1. Different killing mechanisms are used by these two pathogens, although both initially attack by means of surface adhesion.

Killing by means of worm-stars has not been previously described, but aggregation of nematodes to form “Medusa heads” or “rosettes,” resembling worm-stars, has been occasionally reported for microfilariae [21] and soil nematodes [22–24]. These cases may have involved bacterial adhesion and pathogenesis. The ability of larvae to escape by autotomy provides further evidence for the natural occurrence of worm-stars. Predatory trapping by nematophagous soil fungi, which can capture and kill nematodes by means of mechanical traps or adhesive structures, has been much studied [4, 25, 26]. The phenomenon reported here extends the trapping strategy into the bacterial world.

## Experimental Procedures

General *Caenorhabditis* culture conditions were as described in [27].

### Star Formation

Worm-stars were routinely prepared by placing a 0.1 ml drop of washed adult worms in M9 buffer on the plastic lid of a Petri dish and adding 0.05 ml of Verde1 bacteria, grown to stationary phase in LB broth and diluted to 10^8^ cfu/ml. Stars formed more efficiently if worms were grown at low temperature (<20°C).

### Assaying Bacterial Growth

Twelve individual stars of 11–20 worms were allowed to form by transferring young adult worms grown on *E. coli* lawns to unspread NGM plates for 30 min and then picking sets of 25 worms to 0.05 ml drops of 5 × 10^7^ cells/ml of Verde1 in M9 buffer. After 5–10 min, each resulting worm-star was picked to 0.1 ml drops of M9 buffer to wash for 10 min and then picked to individual nutrient-free plates (buffered 1% agarose). Worms in each star were counted. After 0, 15, or 24 hr of incubation at 25°C, stars were separately picked, each to 1 ml M9 buffer in a microfuge tube, ground with sterile sand for 1 minute using a minipestle, vortexed vigorously for 1 min, and diluted for plating on LB plates. Colonies were counted after 24 hours incubation at 30°C, at which point the small Verde1 colonies were easily distinguished from residual large *E. coli* colonies.

## Supplemental Information

Document S1. Figures S1–S4, Tables S1–S4, and Supplemental Experimental Procedures
