# Supplementary material for: Two Leucobacter Strains Exert Complementary Virulence on Caenorhabditis Including Death by Worm-Star Formation
Source: Curr Biol. 2013 Nov 4;23(21):2157–61. doi: 10.1016/j.cub.2013.08.060 (PMC3898767; doi:10.1016/j.cub.2013.08.060)
Supplement: Document S1. Figures S1–S4, Tables S1–S4, and Supplemental Experimental Procedures [file mmc1.pdf]

Current Biology, Volume *23*

Supplemental Information

Two *Leucobacter* Strains Exert Complementary Virulence on *Caenorhabditis* Including Death by Worm-Star Formation

Jonathan Hodgkin, Marie-Anne Félix, Laura C. Clark, Dave Stroud, and Maria J. Gravato-Nobre

Inventory of Supplemental Information

Four Figures and Figure Legends

Figure S1: SEM images of worm infected by Verde1. This relates to Figure 1, allowing higher resolution examination of adhering bacteria.

Figure S2: Verde1 Induces Stars in Multiple Species and in Vegetable Debris. This relates to Figure 2, documenting star formation in other species and in quasi-natural conditions.

Figure S3: Relative Growth of Wild-type and Mutants on Verde1 and Verde2. This relates to Figure 4 and Table S3, illustrating differing levels of population growth.

Figure S4: Induction of *nlp-29* expression. This relates to Figure 4, illustrating up-regulation of a defense gene after Verde1 exposure.

Four Tables

Table S1: Comparison of Leucobacter Strains. This provides documentation of differences between Leucobacter strains.

Table S2: Growth of Caenorhabditis Strains on Different Bacteria. This provides quantitation of growth rates.

Table S3: Growth/Survival of Surface Mutants on Verde1 and Verde2. This documents a range of mutant responses, in addition to the single example illustrated in Figure 4.

Table S4: Effect of Verde2 on Worm-star Formation. This provides documentation of the effect of Verde2 on star formation by Verde1.

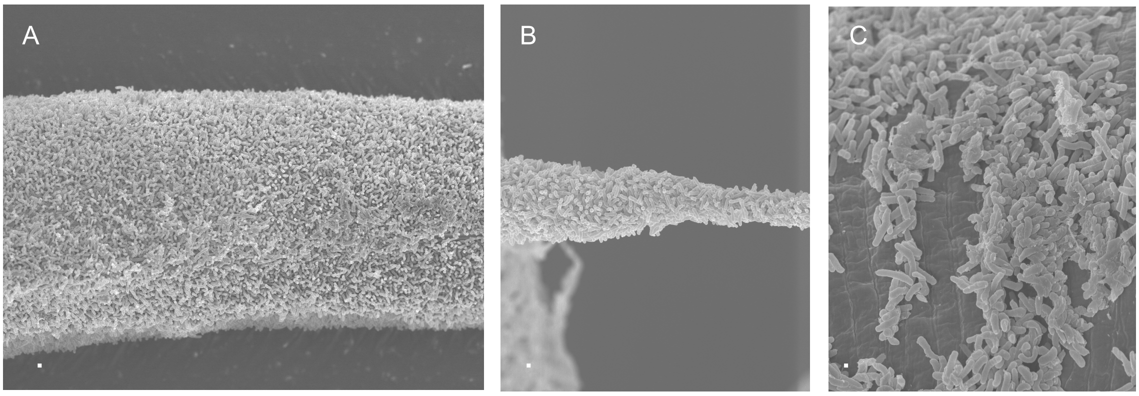

Figure S1: SEM Images of Worms Infected by Verde1.

SEM images of wildtype (N2) worms grown on *E. coli* + Verde1. Lateral views of (A) midbody and (B) tail-spike, showing dense covering of adherent bacteria; scale bar 10μ. (C) Higher magnification view of bacteria adhering to cuticle; circumferential annuli are visible; scale bar 1μ.

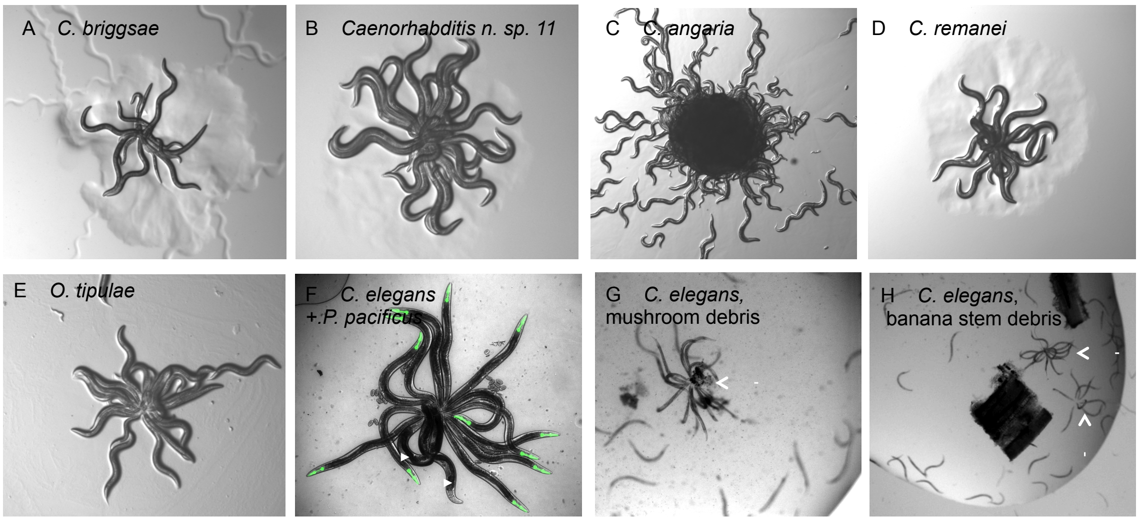

Figure S2: Verde1 Induces Stars in Multiple Species and in Vegetable Debris

Strains illustrated are (A) HK105 (*C. briggsae),* (B) JU1635bl (*Caenorhabditis* n. sp. 11, derived from strain JU1635 by bleach sterilization), (C) PS1010 (*C. angaria*), (D) EM464 (*C. remanei*), (E) CEW1 (*Oscheius tipulae*), (F) CB5584 (*C. elegans,* genotype *mIs12*) + PS312 (*Pristionchus pacificus*); arrowheads mark PS12 individuals, identified by lack of pharyngeal GFP fluorescence, (G) *C. elegans* N2 in liquid mushroom debris, (H) *C. elegans* N2 in liquid banana stem debris; arrows mark incipient worm-stars. Individuals from some examined species, such as *Mesorhabditis longespiculosa* (strain DF5017), failed to form stars either alone or in mixtures with *C. elegans*, but these animals did not swim vigorously in liquid. Similarly, uncoordinated mutants of

C. elegans such as the myosin-defective *unc-54(e190)* failed to be incorporated into stars, even when mixed with excess numbers of wildtype worms.

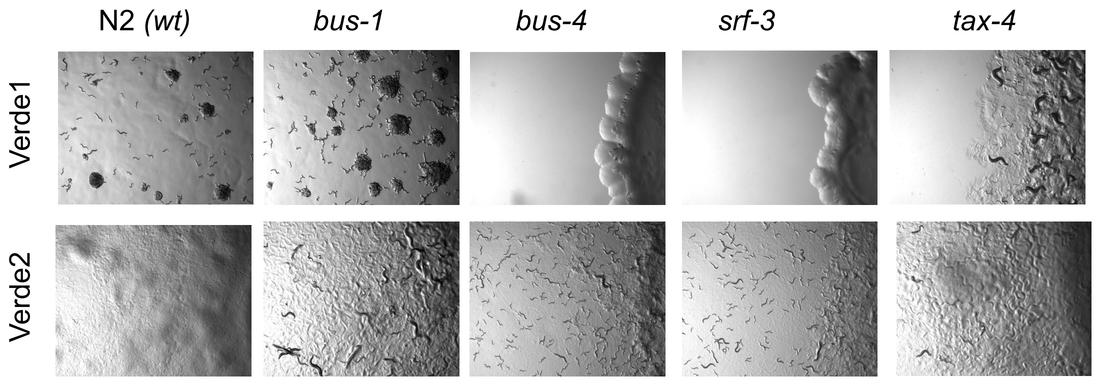

Figure S3: Relative Growth of Mutants on Verde1 and Verde2

Lawns of *E. coli* OP50 + Verde1 or Verde2, 7 days after inoculation with wild-type (N2) or mutant *C. elegans.* Strains are as listed in Table S3.

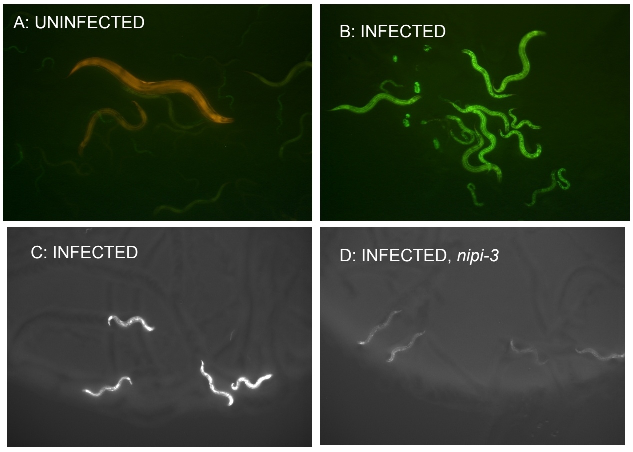

Figure S4: Verde1 Induces a Pathogenic Damage Response.

A population of strain IG274 (*frIs7[nlp-29p::gfp* + *col-12p::DsRed])* growing on *E. coli* (A) and after 24 hours on *E. coli*+Verde1 (B). Green fluorescence indicates induction of the epidermal *nlp-29* promoter. (C, D) Induction of *nlp-29p::gfp* (GFP fluorescence) in worms after 8 hours on *E. coli*+Verde1 in IG274 *(frIs7)* (C) and IG342 *(frIs7; nipi-3)* (D).

Supplemental Tables

Table S1: Comparison of Leucobacter Strains

	NAL101<br>*Leucobacter celer*	CBX151<br>*Leucobacter* Verde1
Oxidase test	Negative	Positive
Growth on L-xylose	+++	-
Assimilation of:		
D-glucose	+	-
D-mannose	+	-
D-maltose	+	-
N-acetylglucosamine	+	-

Table S2: Growth of Caenorhabditis strains on Different Bacteria

Nematode Strain	Bacterial lawn	Dead or arrested	Generation time (hours)	N
JU1635	*E. coli,* V1, V2,??	0	105 ± 7	10
JU1635bl	*E. coli*	0	99 ± 6	10
JU1635bl	*E. coli*, V1	0	143 ± 12	10
JU1635bl	*E. coli,* V2	6	168 ± 12	4
JU1635bl	*E. coli,* V1,V2	10	-	-
N2*	*E. coli*	0	86 ± 4	10
N2*	*E. coli,* V1	0	109 ± 9	10
N2	*E. coli,* V2	2	123 ± 10	8
N2	*E. coli,* V1, V2	3	129 ± 8	7

Table S2 Legend

Lawns (1 cm spots) of *E. coli* (OP50), with added 10% Verde1 (V1) or Verde2 (V2) as indicated, were prepared. 10 late L4 hermaphrodites (<2 hours from lethargus) were picked to separate plates and incubated at 20˚C. The time in hours for the first F1 self-progeny on each plate to reach L4 lethargus was observed. For some worms, all progeny died or arrested prior to the L4 stage, as listed in column 3. Generation time is given as mean ± standard deviation. JU1635 was the original stock sample of *Caenorhabditis* n. sp. 11, which contained unknown bacterial species (??) in addition to Verde1 and Verde2. This stock was bleach-sterilized to yield JU1635bl, which was then propagated on *E. coli* OP50. N2 is the reference wildtype *C. elegans* stock.

*Average brood size for N2 = 335 (n=10) on OP50 versus 302 (n =9) on OP50+Verde1.

Table S3: Growth/Survival of Surface Mutants on Verde1 and Verde2

Genotype	*E. coli* + Verde1	*E. coli* + Verde2
N2 *(wildtype)*	5	0
bus-1(e2678)	5	4
bus-2(e2687)	0	5
bus-3(e2696)	4	2
bus-4(e2693)	0	4
bus-5(e2688)	1	5
bus-6(e2691)	5	4
bus-8(e2698)	1	5
bus-10(e2702)	0	5
bus-12(e2977)	1	5
bus-13(e2710)	1	1
bus-14(e2779)	0	3
bus-15(e2709)	4	0
bus-16(e2802)	1	5
bus-17(e2800)	0	5
bus-18(e2715)	3	5
bus-19(e2964)	1	5
srf-2(jy262)	0	5
srf-3(yj10)	1	5
srf-5(ct115)	0	5
tax-4(e2861)	4	2

Table S3 Legend

Lawns of *E. coli* (OP50) plus 10% Verde1 or Verde2 were prepared and inoculated with 3 L4 hermaphrodites of each genotype. Plates were incubated at 22˚C, and inspected for progeny survival and growth over the next ten days. Scores: 0 (no survival, no long-term growth), 1 (slight larval survival, no long-term growth), 2 (poor survival, some long-term growth), 3 (impaired survival and limited growth), 4 (some impaired survival, reduced long-term growth), 5 (good survival and growth). Representative images, showing lawns after 7 days incubation, are provided in Figure S3. Mutant isolation and properties are described in supplementary references [1] to [6].

Table S4: Effect of Verde2 on Worm-star Formation

Verde1 concentration (x 10^7/ml)	Verde2 concentration (x 10^7/ml)	Time to 50% star formation (minutes)
0	50	-
0	100	-
1	0	9
1	17	6
1	33	3
1	50	4
10	0	5
10	17	3
10	33	2
10	50	2

Table S4 Legend

Verde1 and Verde2 were grown to stationary phase in LB broth, and diluted with M9 buffer. 100 μl drops of pure or mixed bacterial suspensions were prepared, containing the tabled concentrations. 30 adult wildtype hermaphrodite *C. elegans* were added to each drop, and the time taken for 50% or more of these worms to be incorporated into a worm-star was noted.

Verde2 bacteria alone failed to cause star formation, even after incubation for more than an hour.

Supplemental Experimental Procedures

Bacterial growth and strains.

Bacteria were grown to stationary phase in LB broth, at 37˚C (*E. coli)* or 30˚C (Leucobacter strains).

The Leucobacter strains used in this study have been deposited at the DSMZ as: DSM27158 (CBX130), DSM27159 (Verde1/CBX151) and DSM27160 (Verde2/CBX152).

Scanning Electron Microscopy

A nematode culture was resuspended and washed twice in M9 buffer, then fixed overnight in M9buffer + 2.5% glutaraldehyde at 4°C. The fixed animals were rinsed twice in M9 buffer and dehydrated through an ethanol series, pelleting them at 1 g in a tube. The samples were then processed through critical point drying, coated with 20 nm of Au/Pd, and observed using a JEOL 6700F microscope at the Ultrastructural Microscopy Platform of the Pasteur Institute.

SYTO13 labelling

Labelling of infected worms (Figure 1A) was carried out as in [7]. For pre-labelling Verde1, 3 µl of SYTO13 (Invitrogen) 5mM stock in DMSO was diluted to Iml with TBS (10mM Tris pH7.4, 5 mM NaCl, 1mM EDTA). This was mixed with an equal volume of stationary phase LB-grown Verde1 diluted 5-fold with TBS, and the resulting suspension was incubated in the dark for at least 60 minutes at room temperature before using to induce worm-stars (Figure 2F, G).

The dyed bacterial suspension remained competent to induce stars for at least 24 hours.

Fluorescein diacetate labeling

A stock solution of 2 mM fluorescein diacetate in ethanol was diluted 20-fold with M9 buffer, and 10 μl drops added to worm-stars, 5 minutes before mounting the worm-stars for microscopic examination and photography. Freely moving worms exhibited only low levels of fluorescence within gut cells after such staining, and no fluorescence elsewhere in their bodies.

Assaying worms survival on lawns

For data in Figure 4A, 25 μl spots of *E. coli* OP50 plus 10% Verde1 or Verde2 were placed on NGM plates and incubated for 18 hours at 22˚C. For each condition, 4 x 25 synchronized late L4 worms were picked to 4 spots, and plates incubated at 25˚C. Survival was monitored at 4-12 hours intervals; dead worms (motionless and unresponsive to touch) were counted and removed.

Measuring *nlp-29* induction

For reporter gene observations (Figure S5), strains IG274 (genotype *frIs7[nlp-29p::gfp + col-12p::DsRed])* and IG342 (genotype *frIs7; nipi-3(fr4))* were examined. In panels C and D (comparing IG247 and IG342 after 8 hours of exposure to Verde1), identical illumination and camera settings were used, recording via the GFP2 channel on a Leica MZ-FLIII microscope equipped with

a Hamamatsu ORCA-05G camera. The *nipi-3* mutant is defective in the pathogenic induction of *nlp-29* [8].

For qRT-PCR experiments, synchronized L1-stage wildtype animals were grown to the L3/L4 stage on standard NGM plates, then harvested with M9 buffer and transferred to lawns composed of *E. coli* OP50 only or *E. coli* plus 20% Verde1. 0 and 8 hours after challenge, worms were washed off the plates and lysed in Trizol (Invitrogen) to extract total RNA. Purified RNA was DNase treated prior to cDNA synthesis. cDNA was reverse synthesized from 1 µg total RNA (Superscript Vilo kit, Invitrogen) and qPCR was performed using a StepOnePlus Real-time PCR system and SYBR green detection (Applied Biosystems). Gene expression changes were calculated using the comparative Ct (delta delta Ct) method [9]. Experiments were carried out in biological triplicate, and each reaction was performed in 25 µl in technical triplicate. Data were normalized to expression levels of *ama-1*.

Oligonucleotide sequences used for RT-PCR were:

*ama-1*_forw: 5'-CCTACGATGTATCGAGGCAAA-3'

*ama-1*_Rev: 5'-CCTCCCTCCGGTGTAATAATG-3'

*nlp-29*_forw 5'-TATGGAAGAGGATATGGAGGATATG-3'

*nlp-29*_Rev 5'-TCCATGTATTTACTTTCCCCATCC-3'

Decayed vegetable simulations

Small slices (1 cm^2 x 1 mm, ca. 0.1 g) of commercially derived mushroom or banana stem were inoculated with 10^7 Verde1 bacteria and incubated in 3 ml water at 25˚C for 4-6 days before testing for worm-star formation. Testing was

performed by adding approximately 200 adult worms in 0.1 ml water to 0.1 ml of aqueous vegetable debris. Stars began forming within 5 minutes (mushroom debris) or 10 minutes (banana stem debris) (Figure S3G,H). Control samples, prepared without initial inoculation with Verde1 bacteria, failed to induce any stars.

Supplemental References

1. Gravato-Nobre, M. J., Nicholas, H. R., Nijland, R., O'Rourke, D., Whittington, D. E., Yook, K. J., and Hodgkin, J. (2005). Multiple genes affect sensitivity of *Caenorhabditis elegans* to the bacterial pathogen *Microbacterium nematophilum.* Genetics, *171,* 1033-1045.

2. Yook, K., and Hodgkin, J. (2007). Mos1 mutagenesis reveals a diversity of mechanisms affecting response of *Caenorhabditis elegans* to the bacterial pathogen *Microbacterium nematophilum*. Genetics, *175,* 681-697.

3. Gravato-Nobre, M. J., Stroud, D., O'Rourke, D., Darby, C., and Hodgkin, J. (2011). Glycosylation genes expressed in seam cells determine complex surface properties and bacterial adhesion to the cuticle of *Caenorhabditis elegans.* Genetics, *187*, 141-155.

4. Palaima, E., Lemarie, N., Stroud, D., Mizanur, R. M., Hodgkin, J., Gravato-Nobre, M. J., Costello, C. E., and Cipollo, J. F. (2010). The *Caenorhabditis elegans bus-2* mutant reveals a new class of O-glycans affecting bacterial resistance. J Biol Chem, *285,* 17662-17672.

5. Partridge, F. A., Tearle, A. W., Gravato-Nobre, M. J., Schafer, W. R., and Hodgkin, J. (2008). The *C. elegans* glycosyltransferase BUS-8 has two distinct and essential roles in epidermal morphogenesis. Dev Biol, *317*, 549-559.

6. Höflich, J., Berninsone, P., Gobel, C., Gravato-Nobre, M. J., Libby, B. J., Darby, C., Politz, S. M., Hodgkin, J., Hirschberg, C. B., and Baumeister, R. (2004). Loss of *srf-3*-encoded nucleotide sugar transporter activity in *Caenorhabditis elegans* alters surface antigenicity and prevents bacterial adherence. J Biol Chem, *279,* 30440-30448.

7. Hodgkin, J., Kuwabara, P.E., and Corneliussen, B. (2000). A novel bacterial pathogen, *Microbacterium nematophilum*, induces morphological change in the nematode *C. elegans*. Curr. Biol. *10*, 1615-1618.

8. Lee, K. Z., Kniazeva, M., Han, M., Pujol, N., and Ewbank, J. J. (2010) The fatty acid synthase fasn-1 acts upstream of WNK and Ste20/GCK-VI kinases to modulate antimicrobial peptide expression in *C. elegans* epidermis. Virulence 1: 113-122.

9. Schmittgen, T. D., and Livak, J. K. (2008) Analyzing real-time PCR data by the comparative C_T method. Nat. Protoc. *3,* 1101 – 1108.
